# Study of Gene Expression Profiles of Breast Cancers in Indian Women

**DOI:** 10.1038/s41598-019-46261-1

**Published:** 2019-07-10

**Authors:** Shreshtha Malvia, Sarangadhara Appala Raju Bagadi, Dibyabhaba Pradhan, Chintamani Chintamani, Amar Bhatnagar, Deepshikha Arora, Ramesh Sarin, Sunita Saxena

**Affiliations:** 1 Tumour Biology Division, ICMR-National Institute of Pathology, New Delhi, 110029 India; 2 Bioinformatics Cell, ICMR-National Institute of Pathology, New Delhi, 110029 India; 3 Department of Surgery, Safdarjung Hospital, New Delhi, 110029 India; 4 Department of Cancer Surgery, Safdarjung Hospital, New Delhi, 110029 India; 5 Department of Pathology, Indraprastha Apollo Hospital, New Delhi, 110076 India; 6 Department of Surgery, Indraprastha Apollo Hospital, New Delhi, 110076 India

**Keywords:** Breast cancer, Tumour biomarkers

## Abstract

Breast cancer is the most common cancer among women globally. In India, the incidence of breast cancer has increased significantly during the last two decades with a higher proportion of the disease at a young age compared to the west. To understand the molecular processes underlying breast cancer in Indian women, we analysed gene expression profiles of 29 tumours and 9 controls using microarray. In the present study, we obtained 2413 differentially expressed genes, consisting of overexpressed genes such as *COL10A1*, *COL11A1*, *MMP1*, *MMP13*, *MMP11*, *GJB2*, and *CST1* and underexpressed genes such as *PLIN1*, *FABP4*, *LIPE*, *AQP7*, *LEP*, *ADH1A*, *ADH1B*, and *CIDEC*. The deregulated pathways include cell cycle, focal adhesion and metastasis, DNA replication, PPAR signaling, and lipid metabolism. Using PAM50 classifier, we demonstrated the existence of molecular subtypes in Indian women. In addition, qPCR validation of expression of metalloproteinase genes, *MMP1*, *MMP3*, *MMP11*, *MMP13*, *MMP14*, *ADAMTS1*, and *ADAMTS5* showed concordance with that of the microarray data; wherein we found a significant association of *ADAMTS5* down-regulation with older age (≥55 years) of patients. Together, this study reports gene expression profiles of breast tumours from the Indian subcontinent, throwing light on the pathways and genes associated with the breast tumourigenesis in Indian women.

## Introduction

Breast cancer is the most common cancer among women worldwide, representing nearly a quarter (25%) of all cancers with an estimated 2.1 million new cancer cases diagnosed in 2018^1^. Over the past two decades, there has been a rapid increase in breast cancer incidence throughout Asia, mainly South-Eastern Asia, including India^2–6^. Breast cancer is the most common cancer among Indian women in a majority of urban cancer registries at Delhi, Mumbai, Bangalore, Thiruvananthapuram (AAR ranges between 33-41/100000 women) and has rapidly overtaken cervical cancer^7^.

In India, although age-adjusted incidence rate of breast cancer is lower (25.8 per 100 000) than the United States of America (93 per 100 000), age-wise distribution of incidence shows a higher percentage (46.7%) of breast cancer incidence among women below the age of 50 years compared to United States of America (19%)^8^. An incidence rate of 45.5% has been observed in Asian countries for this age group, suggesting a higher incidence of breast cancer in the younger age group in India and other Asian countries as compared to the western population^8^. The underlying causes may be attributed to demographic, genetic, and environmental factors alone or in combination, which may be contributing to the development of the disease at a younger age^9,10^. To our knowledge, there is a single report describing gene expression profiles of breast cancer from Indian patients, focusing mainly on estrogen receptor (ER) positive and ER-negative tumours profiles alone^11^. In the present study, we have analysed the gene signatures and molecular pathways involved in breast carcinogenesis in Indian women by transcriptome profiling.

## Materials and Methods

### Patients and tissue specimen

A total of ninety-seven (97), histologically confirmed breast cancer patients admitted at Safdarjung Hospital or Indraprastha Apollo Hospital, New Delhi, India, during 2008–2012, were enrolled for this study. The study was approved by institutional ethical committees of both Safdarjang Hospital and Indraprastha Apollo Hospital, New Delhi, and informed consent was taken from all the patients. All the experiments were performed following relevant guidelines and regulations. The age of patients, ranged between 25–75 years, comprising of 41 premenopausal and 56 postmenopausal women. The patients were staged according to the American Joint Committee on Cancer (AJCC) guidelines. Expression of ER, progesterone receptor (PR), and human epidermal growth factor receptor 2 (HER2/neu) receptors had been determined by immunohistochemistry (IHC) as described elsewhere^12^. The tumour samples were stratified into, luminal –ER and or PR positive, and HER2/neu negative or ER positive with any PR and HER2/neu positive; basal-ER, PR, HER2/neu negative; and HER2/neu overexpressing tumours-ER, PR negative and HER2/neu positive. Of these enrolled patients, tumour tissue from 77 cases and 38 distant normal breast tissues were used for gene expression profiling and for validation by quantitative reverse transcription PCR (qPCR) (Supplementary Tables S1 and S2). The remaining cases were excluded from the study since they had either received prior therapy or had a history of other malignancies besides breast cancer or had poor RNA quality. All tissue samples were snap frozen in liquid nitrogen immediately after the modified radical mastectomy or after incision/trucut biopsy for RNA isolations and stored in RNA Later (Ambion, Austin, TX) at −80 °C.

### Total RNA extraction

Total RNA was isolated using ‘TRIzol’ reagent (Thermofisher Scientific) following manufacturer’s protocol. In brief, 50–100 mg of tissue samples were pulverized in liquid nitrogen and the powder obtained was lysed using 1 ml ‘TRIzol’, followed by 0.2 ml of chloroform, then, aqueous phase consisting of RNA was separated by centrifugation at 12,000 × g for 15 minutes at 4 °C. RNA was precipitated using an equal volume of isopropanol followed by centrifugation at 12,000 × g for 15 minutes at 4 °C. The RNA pellet was washed with 75% ethanol, air dried and resuspended in 50 μl DEPC treated water. Total RNA isolated from samples was further used for microarray and qPCR. The RNA samples were treated with DNase I (Qiagen, Hilden, Germany) and purified on RNeasy mini column (Qiagen, Hilden, Germany) before using for experiments to avoid genomic DNA contamination. In brief, after adjusting sample volume to 100 μl, 350 μl RLT buffer and 250 μl of absolute ethanol were added to it, the mixture was placed onto the column, 10 μl of DNase was added to the column for 30 minutes duration at room temperature, followed by two washes with 500 μl of buffer RPE and centrifugation at 10,000 rpm for 1 minute. Total RNA was eluted using 30 μl of RNase free water followed by centrifugation at 10,000 rpm for 3 minutes. Quantity and quality of the purified RNA were determined by Nanodrop (Thermofischer Scientific, U.S.A) and Agilent 2100 Bioanalyzer (Agilent Technologies, Santa Clara, California) respectively. Tumour tissues with RNA integrity number (RIN) ≥7 were included in the study, in the case of controls, all the samples had RIN ≥7 except for two controls which had RIN of 6.8 and 6.1 respectively.

### Gene expression profiling by microarray

Whole genome-wide expression profiling was done using HumanWG-6 v3.0 and HumanHT-12 v3 direct hybridization assay (Illumina, San Diego, CA) in 2 batches. The HumanWG-6 Bead Chip contains >48,000 probes, while HumanHT-12 chip contains the same panel of probes targeting more than 25,000 (human) genes from Reference Sequence (RefSeq) and UniGene database, from the National Center for Biotechnology Information (NCBI); but the later chip provides higher throughput processing of 12 samples per chip. In the present study a total of 29 tumour samples, including 12 Early-onset tumours (ET), from patients having age ≤40 years, and 17 Late-onset tumours (LT), from patients having age ≥55 years, along with 9 distant normal specimens as controls were used for gene expression profiling. Five hundred nanograms of total RNA was converted to complementary DNA (cDNA), followed by an *in vitro* transcription step to generate labeled complementary RNA(cRNA) using the ‘Ambion Illumina Total Prep RNA Amplification Kit’ (Ambion, Austin, TX) as per manufacturer’s instructions. The labeled cRNA was hybridized to bead chip array and washed following manufacturer’s protocols, which was scanned by ‘Illumina Bead Array Reader, to obtain the raw data. The expression profiles of 29 cases and 9 controls have been deposited in NCBI’s Gene Expression Omnibus (GEO) with GSE accession number GSE 89116.

### Microarray data analysis

Raw data generated from the scanned slides was subjected to background correction followed by log2 transformation on Illumina’s Genome Studio software. The data was quantile normalized using Linear Models for Microarray Data (LIMMA- v.3.36.1)^13^ on Bioconductor package R.3.3.2^14^. Further, differentially expressed genes (DEGs) in Total tumours (TT), Early-onset tumours (ET) and Late-onset tumours (LT) compared to normal controls; and in various molecular subgroups viz. luminal, basal, HER2/neu were obtained using LIMMA. Molecular subtypes were predicted using ‘molecular.subtyping’ function on ‘Genefu’^15^ (v.2.12.0) using R as explained for ‘Compare Molecular Subtype Classifications’, in the ‘Genefu’ manual (bioconductor.org).Expression profiles obtained in the present study were compared with that of the western population, reported by Clarke *et al*.^16^ and Maubant *et al*.^17^ (GSE42568 and GSE65194 from the NCBI Gene Expression Omnibus).

### Hierarchical clustering/gene ontology (GO) and network analysis

Unsupervised hierarchical clustering was performed for the DEGs by Cluster 3^18^ software. The normalized probe intensities were median centered, Pearson correlation was used for similarity/distance measurement and centroid linkage clustering was performed. Further, JavaTree View Software^19^ was used to view the clustering image. Gene ontology analysis was performed using Pathway Express software^20^ (from Onto tools). Kyoto Encyclopedia of Genes and Genomes (KEGG) database^21^ was used to determine specific pathways pertaining to differentially expressed genes. Further, Gene Set enrichment analysis (GSEA)^22^ software was used to gain insights into the top 50 DEGs and also to generate a heatmap. Network analysis was done using ‘NetworkAnalyst’^23^, where protein-protein interactions networks were predicted using search tool for Recurring Instances of Neighboring Genes (STRING)^24^.

### Validation of differential gene expression by qPCR

Quantitative reverse transcription PCR was done for *MMP1*, *MMP3*, *MMP11*, *MMP13*, *MMP14*, *ADAMTS1*, *ADAMTS5*, *18sRNA*, *β-actin*, and *PSMC4* genes, where *18sRNA*, *β-actin*, and *PSMC4* were used as endogenous controls for normalization of the qPCR data. QPCR validation of the above genes was done in 67 of the 77 tumours selected (depending on the availability of RNA) for the present study and 38 distant normal tissues along with 2 human mammary total RNA (Ambion, Austin, TX) (Supplementary Tables S1). The total RNA from the 38 distant normal tissues were combined to a single pool, which along with the 2 human mammary total RNAs (obtained from Ambion) were used as controls for determining the expression by qPCR.

The reverse transcription (RT) reaction was carried out using 1 μg of total RNA, random primers, and SuperScript III RT (Invitrogen, Thermo Fisher Scientific) at 50 °C for 50 minutes in a total reaction volume of 20 μL following the manufacturer’s protocol. The cDNA generated by RT was diluted 5 folds and qPCR was carried out using 4.5 μL of the diluted cDNA, 5.0 μL of SYBR green mix (2X) and 0.25 pm of gene-specific primers in a total volume of 10 μL reaction. A non-RT control was also used during qPCRs to ensure lack of nonspecific amplification due to genomic DNA contamination. Most of the qPCR primers were designed (primer quest tool from Integrative DNA Technologies) such that they span exon junctions (except for *MMP11*, *MMP13*, *ADAMTS5*, and *ACTINB* genes) to avoid nonspecific amplification (Supplementary Table S3). The following cycling conditions were used for qPCRs, initial denaturation at 95 °C for 2 minutes, 40 cycles of 95 °C for 10 seconds and 60 °C for 1 minute on the StepOne Real-time PCR (ABI, Foster City, CA). All the samples were run in triplicate in a 96 well plate (ABI, Foster City, CA). The specificity of all the primers was confirmed by melting curve analysis on StepOne software v.2.3 (ABI, Foster City, CA). The mean Ct obtained for each gene was normalized with endogenous controls to obtain ΔCt, and further fold change (FC) was obtained by the ΔΔCt method on Data assist software v.3.01 (ABI, Foster City, CA).

### Statistical analysis

Identification of differentially expressed genes (DEGs) was done by fitting gene-wise linear models to the gene expression data obtained from microarray experiments; the ‘lmFit’ function was used on LIMMA^13^ for different groups (TT, ET, and LT compared to controls). False discovery rate was controlled by Benjamini Hochberg FDR^25^ correction for a significant p-value cutoff of 0.05 and fold change ≥±1.5. LIMMA was also used for determining DEGs (fold change ≥±2.0 and p-value ≤ 0.05) from the western datasets, and for comparison with our data. Mann-Whitney U test was used to determine differential expression of MMP genes and their association with various clinicopathological parameters.

## Results

Among 77 histopathologically confirmed cases of breast cancer enrolled for this study, 35 cases (45.45%) were below 40 years of age (ET) while, 42 cases (54.54%) were above 55 years of age (LT) (Supplementary Table S1). The tumours were staged into, stage I having 2 cases (2.59%), stage II having 32 cases (41.5%), stage III having 37 cases (48.05%) and stage IV having 3 cases (3.89%), while for 3 cases (3.89%) stage is not known. Molecular subtyping based on expression of ER, PR, and HER2/neu, yielded 33 cases (42.85%) to luminal subtype, 17 (22.07%) cases to basal subtype, and 23 cases (29.87%) to HER2/neu overexpressing subtype (Supplementary Table S2), and for 4 cases (5.19%) molecular subtype could not be determined as the expression of ER, PR and HER2/neu were not available for these cases.

### Microarray analysis

**Figure 1:**
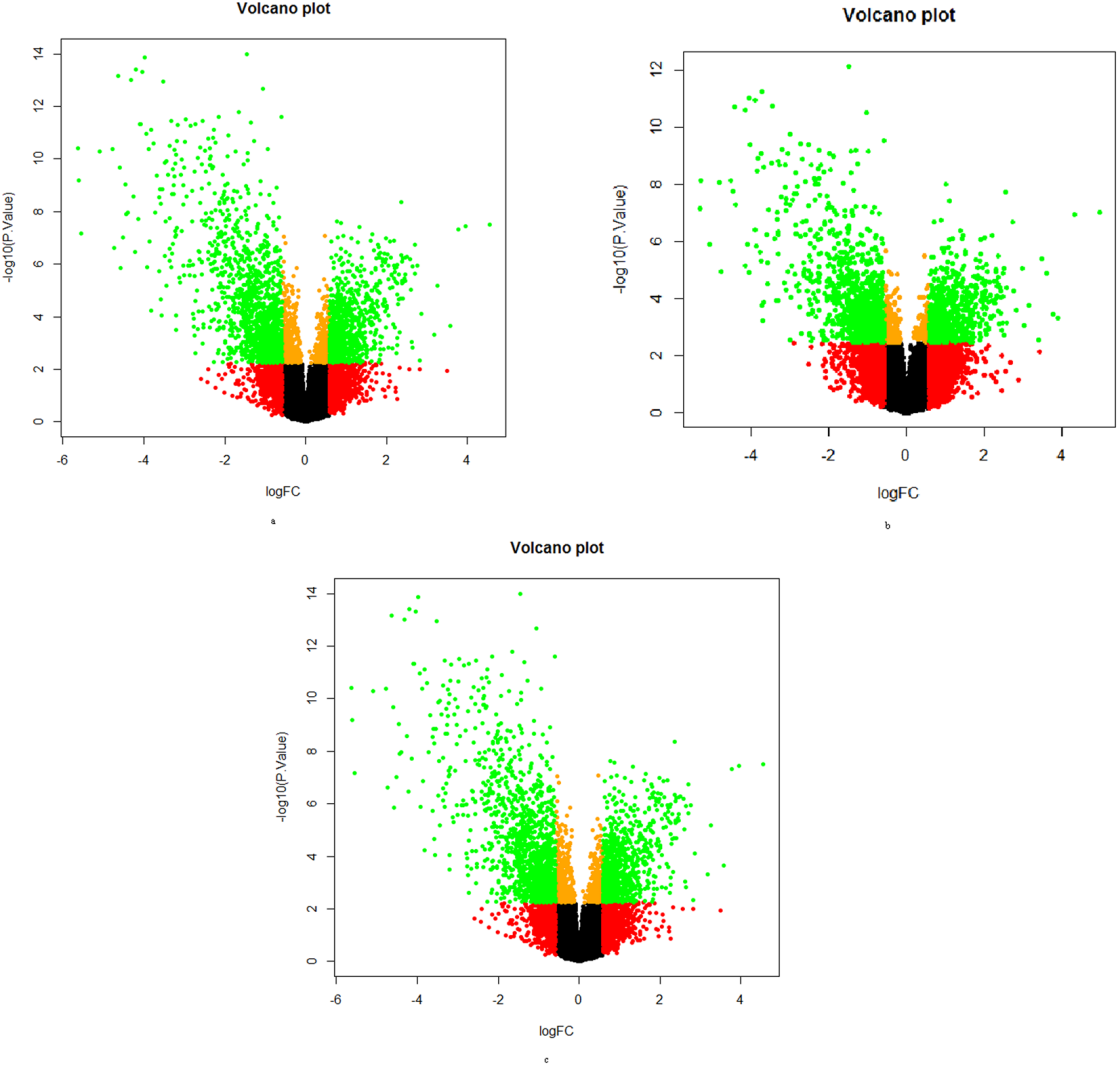
Volcano plots showing the distribution of gene expression by microarray in total breast tumours and early- and late-onset breast tumours as compared to controls. The plot shows gene expression profiles of breast tumours. The plot was obtained between negative log p-value (y-axis) and log fold change (x-axis). Each dot represents one gene, genes shown in green colour had significant fold change (FC ≥ 1.5, and adjusted p ≤ 0.05) while the remaining genes depicted in red, black and orange colour didn’t reach significance. (**a**) Plot shows gene expression profiles of total tumours vs controls (**b**) Plot shows the gene expression profiles of early-onset tumours vs controls (**c**) Plot shows the gene expression profiles of late-onset tumours vs controls.

Genome-wide expression profiling was done in 29 tumours, and 9 distant histologically confirmed normal control tissues, in the present study. The raw data obtained was analysed using LIMMA, a cutoff of FC ≥ 1.5 and p-value ≤ 0.05 were used for identification of differentially expressed genes. Volcano plots were drawn to get an overview of differential gene expression among different tumour groups (Fig. 1); it was observed that the proportion of down-regulated genes were more than up-regulated genes in TT, ET, and LT tumour groups.

### Differential gene expression analysis

A total of 2413 differentially expressed genes (DEGs), including 991 up-regulated genes and 1422 down-regulated genes (Supplementary Table S4), were found in breast tumours (TT) compared to controls. The top up-regulated genes include *COL10A1*, *MMP11*, *GJB2*, *CST1*, *KIAA1199*, *MMP1*, *MMP13*, *CEACAM6*, *BUB1* and *ASPM* involved in cell cycle, focal adhesion and metastasis, while top down-regulated genes were *PLIN*, *KIAA1881*, *ADH1A*, *ADH1B*, *CIDEC*, *THRSP*, *GPD1*, *TIMP4*, *FABP4* and *C7* involved in lipid metabolism, and PPAR pathway (Table 1).

**Table 1:** Top ten up-regulated and down-regulated genes between breast tumours and controls.

GENE	(FC)	Adjusted.p-value	Accession
**Up-regulated**			
*COL10A1*	23.2815	5.96E-06	NM_000493.2
*MMP11*	15.45492	6.55E-06	NM_005940.3
*GJB2*	13.68954	8.27E-06	NM_004004.3
*CST1*	11.94889	0.004912	NM_001898.2
*KIAA1199*	9.509539	0.000351	NM_018689.1
*MMP1*	9.096327	0.008953	NM_002421.2
*MMP13*	7.297567	0.002203	NM_002427.2
*CEACAM6*	7.014203	0.042617	NM_002483.3
*BUB1*	6.795974	9.11E-05	NM_004336.2
*ASPM*	6.500159	2.39E-05	NM_018136.2
**Down-regulated**			
*C7*	−22.871	1.39E-05	NM_000587.2
*FABP4*	−23.8769	0.000104	NM_001442.1
*TIMP4*	−24.1717	1.03E-07	NM_003256.2
*GPD1*	−24.9122	3.83E-10	NM_005276.2
*THRSP*	−26.7033	3.01E-05	NM_003251.2
*CIDEC*	−27.4842	3.18E-08	NM_022094.2
*ADH1B*	−33.9347	3.6E-08	NM_000668.3
*ADH1A*	−46.9269	1.12E-05	NM_000667.2
*KIAA1881*	−48.6453	2.71E-07	Hs.567652

### Hierarchical clustering and gene ontology

**Figure 2:**
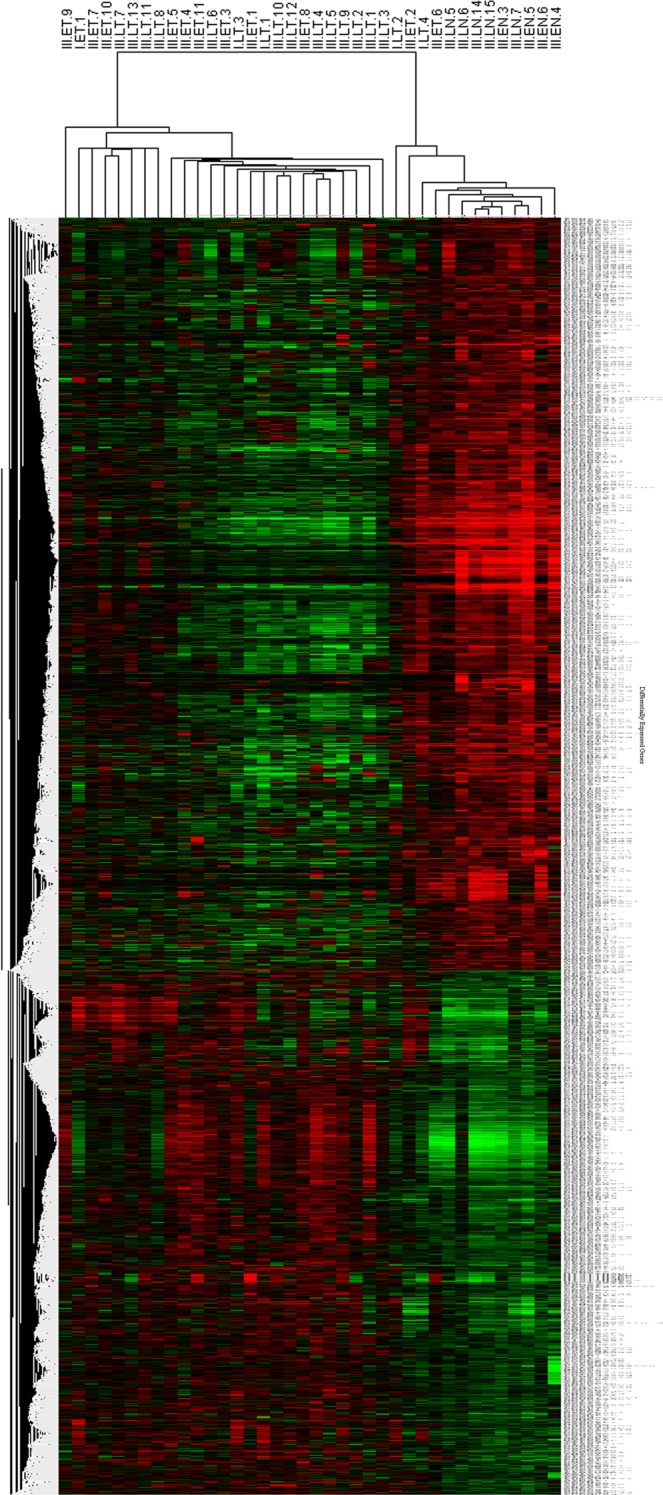
Unsupervised hierarchical clustering of differentially expressed genes. Heatmap showing the hierarchical clustering of tumours based on their gene expression. 2413 genes were found to be differentially expressed in tumours (FC ≥ 1.5, and adjusted p-value ≤ 0.05) forming distinct up-regulated and dow-nregulated clusters. Red colour represents up-regulation and green colour represents down-regulation. The differentially expressed genes are mentioned on the y-axis, and sample IDs are mentioned on the x-axis.

Unsupervised hierarchal clustering of the DEGs in breast tumours (TT), yielded two distinct clusters of up and down-regulated genes (Fig. 2, Supplementary Fig. S1). The topmost 50 genes were clustered separately for better visualisation of data (Supplementary Fig. S2). Further, pathway analysis was done to identify the biological pathways associated with breast cancer. The major pathways found to be deregulated include cell adhesion molecules, cell cycle, adherens junction, PPAR signalling, complement and coagulation cascades, focal adhesion, ECM-receptor interaction, DNA replication, adipocytokine signaling, pathways in cancer (Table 2). The pathways associated with up-regulated genes include cell cycle, systemic lupus erythematosus, DNA Replication, ECM-receptor interaction, p53 signalling (Supplementary Table S5) while the pathways associated with down-regulated genes include leukocyte transendothelial migration, cell adhesion molecules, adherens junction, complement and coagulation cascade, PPAR signaling, circadian rhythm, focal adhesion, adipocytokine signaling pathway, and tight junction (Supplementary Table S6).

**Table 2:** Gene ontology analysis of DEGs found in the breast tumours.

Rank	Pathway Name	Impact Factor	Input Genes in Pathway	List of Genes	Corrected p-value
1	Cell adhesion molecules (CAMs)	235.367	21	*CADM3*, *CLDN15*, *CDH5*, *CLDN11*, *CLDN5*, *ESAM*, *ICAM2*, *JAM2*, *NLGN1*, *PECAM1*, *PTPRM*, *PVRL3*, *SELP*, *PVRL2*, *CD34*, *ITGA9*, *C10ORF54*, *ITGA7*, *LAMA2*, *LAMA3* and *NEGR1*	0.011143
2	Cell cycle	22.121	34	*E2F2*, *E2F3*, *CDC2*, *E2F5*, *CDCA5*, *PKMYT1*, *CDC45L*, *ORC1L*, *CHEK1*, *PTTG1*, *CCNE2*, *CCNE1*, *RAD21*, *MCM7*, *CDKN2C*, *BUB1*, *CCNA2*, *MYC*, *CDCA5*, *ESPL1*, *CDC20*, *MCM2*, *CDC25C*, *MCM4*, *CDC25A*, *MCM6*, *CDKN1C*, *CCNB1*, *CCNB2*, *MAD2L1*, *TTK*, *PLK1*, *GSK3B*, *and BUB1B*	8.12E-10
3	Adherens junction	20.559	15	*LEF1*, *PTPRB*, *PTPRM*, *WASF2*, *SORBS1*, *SSX2IP*, *PVRL3*, *PVRL2*, *PVRL4*, *CDH5*, *PRR4*, *CADM3*, *ANG*, *TCF7L2*, and *CDH23*	0.00458
4	PPAR signalling pathway	15.598	20	*ACADL*, *ACSL1*, *ADIPOQ*, *ANGPTL4*, *AQP7*, *CD36*, *EHHADH*, *FABP4*, *GK*, *LPL*, *MMP1*, *OLR1*, *PCK1*, *PLIN*, *PLTP*, *PPARG*, *SORBS1*, *RXRA*, *PPARGC1A*, and *NR1H3*	3.54E-06
5	Complement and coagulation cascades	15.474	19	*F12*, *C7*, *A2M*, *F10*, *F13A1*, *C6*, *SERPING1*, *C4BPA*, *PLAUR*, *VWF*, *THBD*, *SERPINF2*, *F3*, *CD59*, *CFH*, *TFPI*, *CFI*, *CFD*, and *PROS1*	1.40E-05
6	Focal adhesion	14.939	40	*COL3A1*, *ITGA11*, *LOXL4*, *CAV2*, *MYL7*, *CAV1*, *TLN2*, *FIGF*, *PXN*, *MYL9*, *LAMB2*, *ARHGAP5*, *TNN*, *PDGFD*, *COL11A1*, *SPP1*, *PIK3R2*, *THBS4*, *FN1*, *BRAF*, *IGF1*, *FLNC*, *COL5A2*, *COL5A1*, *LAMA2*, *VWF*, *VEGFC*, *ITGA9*, *LAMA4*, *PPP1CA*, *LAMA3*, *CCND2*, *JUN*, *GSK3B*, *ITGA7*, *COL1A2*, *RELN*, *COL1A1*, *COL24A1*, and *MYLK*	5.09E-06
7	ECM-receptor interaction	14.846	23	*IBSP*, *COL3A1*, *ITGA11*, *COL5A2*, *COL5A1*, *HMMR*, *LAMA2*, *VWF*, *LAMA4*, *LAMB2*, *CD36*, *LAMA3*, *ITGA7*, *COL1A2*, *RELN*, *TNN*, *SV2B*, *COL1A1*, *COL24A1*, *COL11A1*, *SPP1*, *FN1*, and *THBS4*	1.69E-06
8	DNA replication	10.812	12	*DNA2*, *RFC4*, *MCM7*, *POLE2*, *RFC2*, *PRIM2*, *MCM2*, *POLA2*, *RNASEH2A*, *MCM4*, *FEN1*, and *MCM6*	4.31E-05
9	Adipocytokine signaling pathway	8.699	13	*LEP*, *IRS2*, *ACSL1*, *CD36*, *SOCS3*, *LEPR*, *RXRA*, *ACACB*, *POMC*, *ADIPOQ*, *PPARGC1A*, *SLC2A1*, and *PCK1*	0.010329
10	Pathways in cancer	9.026	50	*ACVR1C*, *E2F2*, *E2F3*, *FGF7*, *STAT5A*, *SLC2A1*, *STAT5B*, *PPARG*, *MYC*, *FGF2*, *MMP1*,*CCNE2*, *WNT2*, *FOS*, *FIGF*, *CCNE1*, *TCF7L1*, *WNT11*, *LEF1*, *DAPK2*, *KIT*, *ZBTB16*, *IL6*, *BRAF*, *RXRA*, *FZD5*, *CEBPA*, *BMP2*, *VEGFC*, *EVI1*, *JUN*, *EPAS1*, *GNAI1*, *FOXO1*, *FN1*, *TGFBR2*, *TCF7L2*, *FZD4*, *LAMB2*, *LAMA2*, *PIK3R2*, *IGF1*, *BIRC5*, *LAMA4*, *LAMA3*, *GSK3B*, *SMAD3*, *NRAS*, *WNT7B*, and *BAX*	6.97E-04

### Gene networking analysis

**Figure 3:**
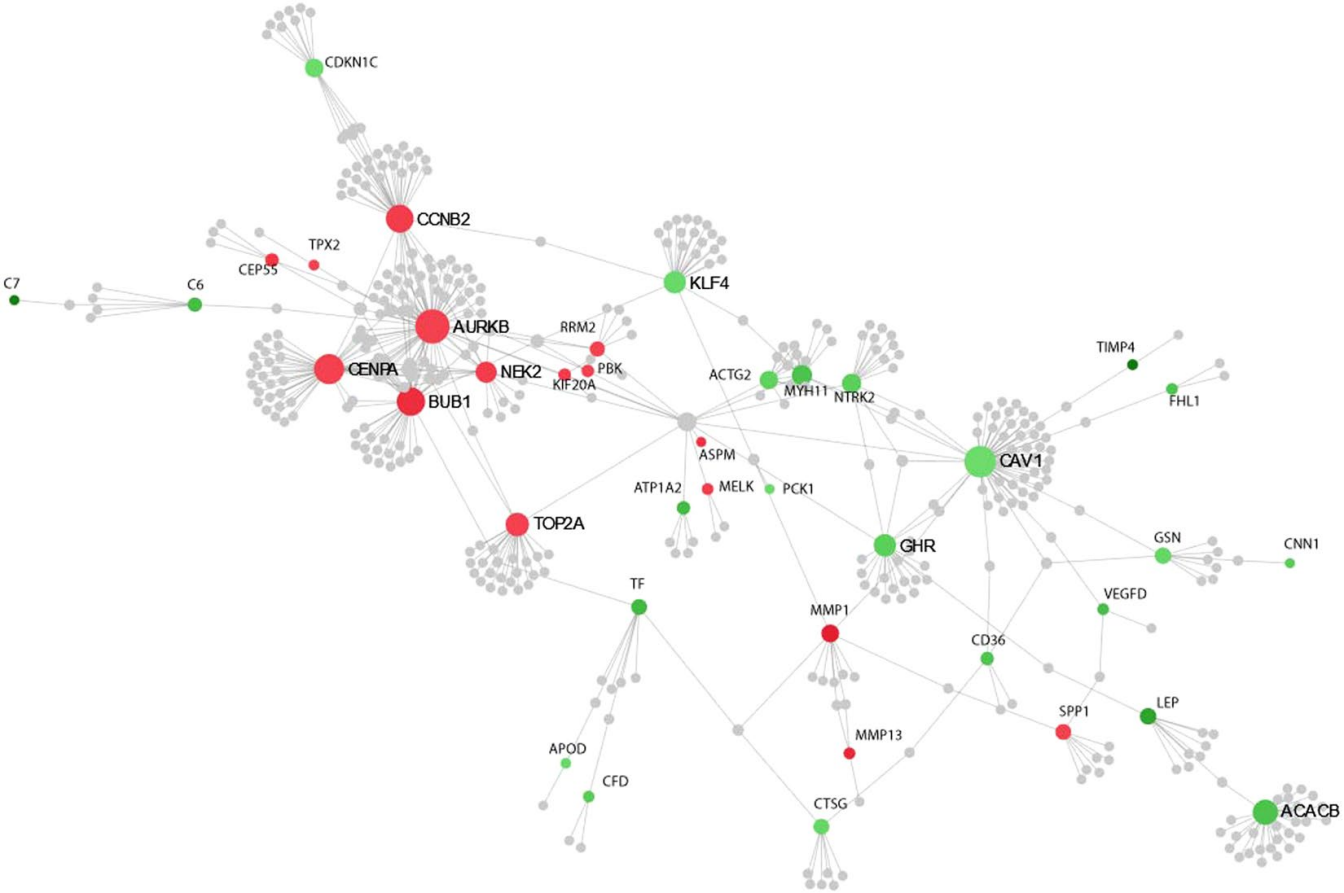
Showing gene network analysis of differentially expressed genes in breast tumours. Interactive gene networks were identified based on their position in the network by two measures; degree centrality, where the importance of a node is dependent on the number of connections to other nodes and betweenness centrality, which measures the number of shortest paths going through a node. Nodes with a higher degree are hubs of the network, and the size of the nodes is based on their degree values, with a bigger size accounting for larger degree values. The colour of the nodes is related to the expression of genes, where up-regulated nodes are shown in red and down-regulated nodes in green colour while the grey coloured nodes are those genes that are not present in our data set but are part of the PPI network (The network analysis was done with DEGs having FC ≥±5). Among the gene networks, *AURKB*, *CENPA*, *TOP2A*, *BUB1*, *CCNB2*, *MMP1*, and *SPP1* were the most interactive nodes.

Gene network analysis was performed to identify the key regulatory genes among the DEGs found in the breast tumours (TTs). The topmost interactive up-regulated nodes include, *AURKB*, *CENPA*, *TOP2A*, *BUB1*, *CCNB2*, *MMP1*, and *SPP1* genes involved in cancer metastasis, cell cycle, and mitosis; and the down-regulated nodes include *CAV1*, *ACACB*, *NTRK2*, *KLF4,* and *MYH11* genes involved in regulation of Ras-ERK, fatty acid synthesis, MAP kinase and JAK2/STAT3/5, and ATP hydrolysis pathways (Fig. 3).

### Comparison of DEGs between Indian and western patients

Gene expression signatures found in the present study were further compared with that of the gene expression profiles of breast tumours derived from the western population (GSE 65194 and GSE 42568). A total of 5062 DEGs, including 3789 up-regulated and 1273 down-regulated genes were found with western data set. Comparison of DEGs between Indian patients (found in the present study) with that of western, showed 715 genes (Supplementary Table S7) that are common between both the data sets, while 558 DEGs were associated with Indian patients (Supplementary Table S8). Pathway analysis of common DEGs among the two population showed deregulation of leukocyte transendothelial migration (*ESAM*, and *MYL7)*, cytokine receptor interaction (*IL17B*, *CNTFR*, *FIGF*, *MPL*, and *CCL21)*, and adherens junction (*PVRL3*, *PVRL4*, and *TCF7l1*) pathway.

### DEGs in early- and late-onset breast cancer

Analysis of DEGs amongst ET and LT groups showed 1685 DEGs in ET and 2379 DEGs in LT compared to controls (Table 3, Supplementary Tables S9 and S10). When DEGs between ET and LT were compared, a majority of common and few uniquely expressed genes were found between the two groups; though there was a difference in terms of fold expression of the genes. When we used ANOVA to identify genes significantly associated with ET and LT, 420 genes were found to be associated with ET, 1114 genes were found to be associated with LT while, 1265 genes were common between both the groups (Supplementary Fig. S3). Pathways analysis of the DEGs revealed the involvement of similar pathways in ET and LT groups (Supplementary Tables S11 and S12) but, the genes associated with each of these pathways were found to be different. Cellular processes such as leukocyte transendothelial migration, cell adhesion molecules, PPAR signaling, cell cycle, ECM-receptor interaction pathways are some of the pathways that were altered in ET and LT. Networking analysis in ET shown *BRAF* gene as a top overexpressed node while *SMAD3* gene was an top underexpressed node. The top nodes getting up-regulated exclusively in LT were *MCM2*, and *MAD2L1*, while *PXN*, and *SOCS3* were top down-regulated nodes.

**Table 3:** Top ten up-regulated and down-regulated genes between (a) Early-onset breast tumours and controls and (b) Late -onset- tumours and controls.

Genes	(FC)_Early-onset tumours	Adjusted p-value	Accession
(**a**) **Top 10 genes in early-onset tumours**			
**Up-regulated**			
*COL10A1*	31.47292482	1.46E-07	NM_000493.2
*MMP11*	20.16883881	1.62E-07	NM_005940.3
*CST1*	15.09417634	0.000998	NM_001898.2
*MMP1*	13.78096344	0.001196	NM_002421.2
*KIAA1199*	12.24468581	2.69E-05	NM_018689.1
*GJB2*	11.13842735	2.76E-07	NM_004004.3
*S100P*	10.67661335	0.011113	NM_005980.2
*MMP13*	8.92393836	0.000343	NM_002427.2
*PITX1*	8.241605007	0.00272	NM_002653.3
*TUBB3*	7.974669152	2.51E-05	NM_006086.2
**Down-regulated**			
*ALDH1L1*	−17.95091861	1.08E-12	NM_012190.2
*G0S2*	−21.35394129	9.76E-09	NM_015714.2
*GPD1*	−21.60426989	6.70E-13	NM_005276.2
*TIMP4*	−22.50528229	2.24E-09	NM_003256.2
*CIDEC*	−23.24428146	3.64E-10	NM_022094.2
*ADH1A*	−27.50733965	1.64E-07	NM_000667.2
*ADH1B*	−28.39338049	4.42E-10	NM_000668.3
*THRSP*	−33.98850427	1.47E-06	NM_003251.2
*PLIN*	−39.64517112	2.95E-10	NM_002666.3
*KIAA1881*	−40.50151762	5.61E-09	Hs.567652
**(b) Top 10 genes in late-onset tumours**			
**Up-regulated**			
*COL10A1*	18.81883032	1.46E-07	NM_000493.2
*GJB2*	15.83475077	2.76E-07	NM_004004.3
*MMP11*	12.80727039	1.62E-07	NM_005940.3
*CST1*	10.13195192	0.000998	NM_001898.2
*CEACAM6*	8.845061876	0.012935	NM_002483.3
*KIAA1199*	7.955397998	2.69E-05	NM_018689.1
*BUB1*	7.409286293	6.92E-06	NM_004336.2
*ASPM*	7.250017904	1.01E-06	NM_018136.2
*FAM83D*	7.144854585	1.29E-05	NM_030919.1
*CKAP2L*	6.929207833	6.64E-06	NM_152515.2
**Down-regulated**			
*TIMP4*	−25.42177657	2.24E-09	NM_003256.2
*C7*	−27.25711853	5.32E-07	NM_000587.2
*GPD1*	−27.54770546	6.70E-13	NM_005276.2
*C2ORF40*	−28.62109977	2.00E-08	NM_032411.1
*CIDEC*	−30.93481391	3.64E-10	NM_022094.2
*FABP4*	−36.04296806	1.88E-06	NM_001442.1
*ADH1B*	−38.48568016	4.42E-10	NM_000668.3
*KIAA1881*	−55.36142413	5.61E-09	Hs.567652
*PLIN*	−58.36652422	2.95E-10	NM_002666.3
*ADH1A*	−68.41751514	1.64E-07	NM_000667.2

### DEGs in various stages of breast cancer

We analysed DEGs between lower stage (stage I and II) and advanced stage (stage III and IV) tumours, where 1386 DEGs associated with lower stage (stage I and II) and 200 DEGs associated with advanced stage (stage III and IV), and 1336 DEGs common between both the groups were found (Supplementary Tables S13 and S14). Gene ontology analysis of these DEGs showed that in advanced stage tumours, pathways such as cell adhesion (*VCAN*), ECM receptor interaction (*COL1A2*, *COL3A1*, *ITGA11*, and *TNN*) and pathways in cancer (*JUN*, and *MYC*) getting deregulated.

### Molecular subtypes

**Figure 4:**
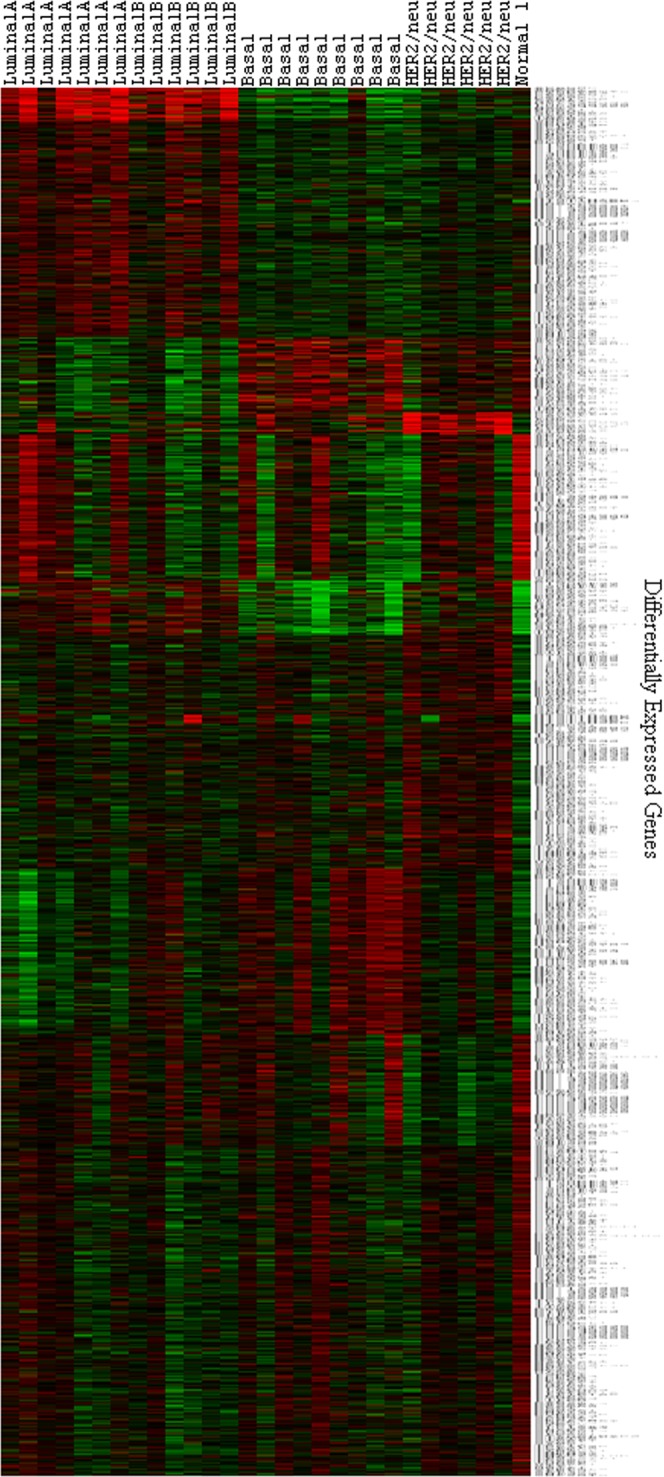
Heatmap showing hierarchical clustering of predicted molecular subtypes. Molecular subtypes were predicted using PAM50 classifier in breast tumours, consisting of subtypes viz. luminal A, luminal B, HER2/neu, basal and normal-like (FC ≥ 1.5, and adjusted p-value ≤ 0.05). Genes pertaining to each subtype formed distinct clusters. Red colour represents up-regulation and green colour depicts down-regulation. The subtypes are mentioned on x-axis while differentially expressed genes are mentioned on the y-axis.

**Figure 5:**
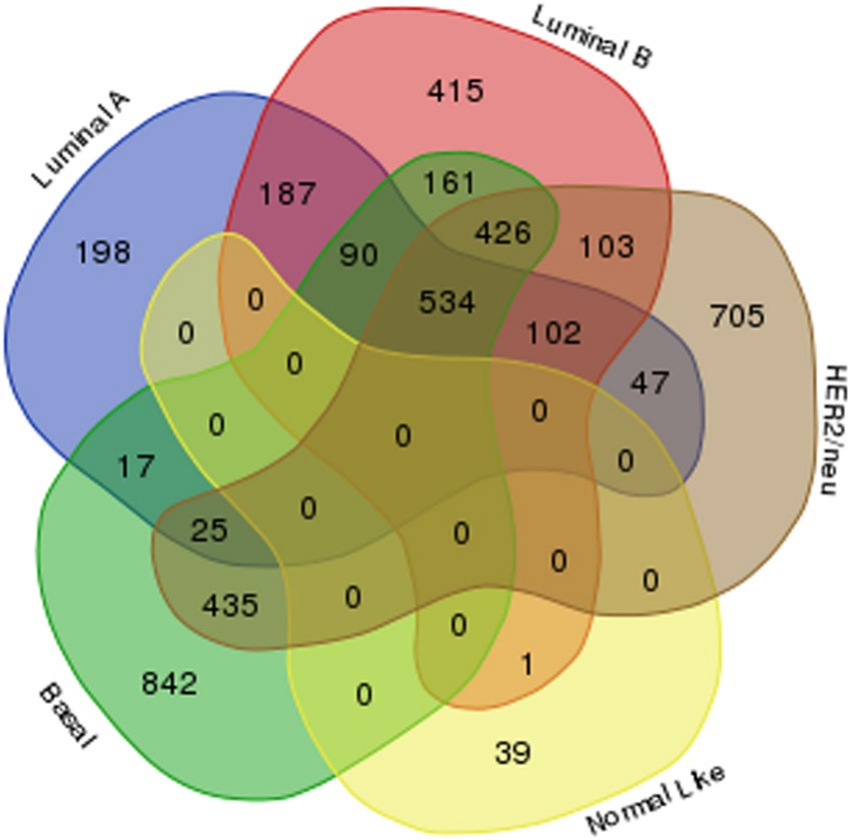
Venn diagram showing the common and unique genes belonging to each molecular subtypes in breast tumours. Venn diagram showing differentially expressed genes unique in basal subtype (842) followed by HER2/neu (705), luminal B & A (415, 198) and normal-like subtypes (39).

Molecular subtyping based on gene expression profiles was done using Prediction Analysis of Microarray 50 (PAM50) classifier. Using ‘Genefu’ we could stratify tumour samples into, luminal-A consisting of 7 cases (24%), luminal-B consisting of 6 cases (20%), basal consisting of 9 cases (31%), HER2/neu overexpressing tumours consisting of 6 cases (20%) and normal-like subtype consisting of 1 case (3%). Further, unsupervised hierarchical clustering of PAM 50 genes yielded distinct gene clusters corresponding to each molecular subtypes (Fig. 4, Supplementary Fig. S4). This confirms the existence of molecular subtypes in breast tumours of Indian women, similar to that reported in the western population. In addition, we obtained DEGs associated with each subtype (Supplementary Table S15) consisting of 198, 415, 842, 705 and 39 (Fig. 5) genes unique to luminal-A, luminal-B, basal, HER2/neu overexpressing and normal-like subtypes respectively.

### Validation of gene expression profiles

**Figure 6:**
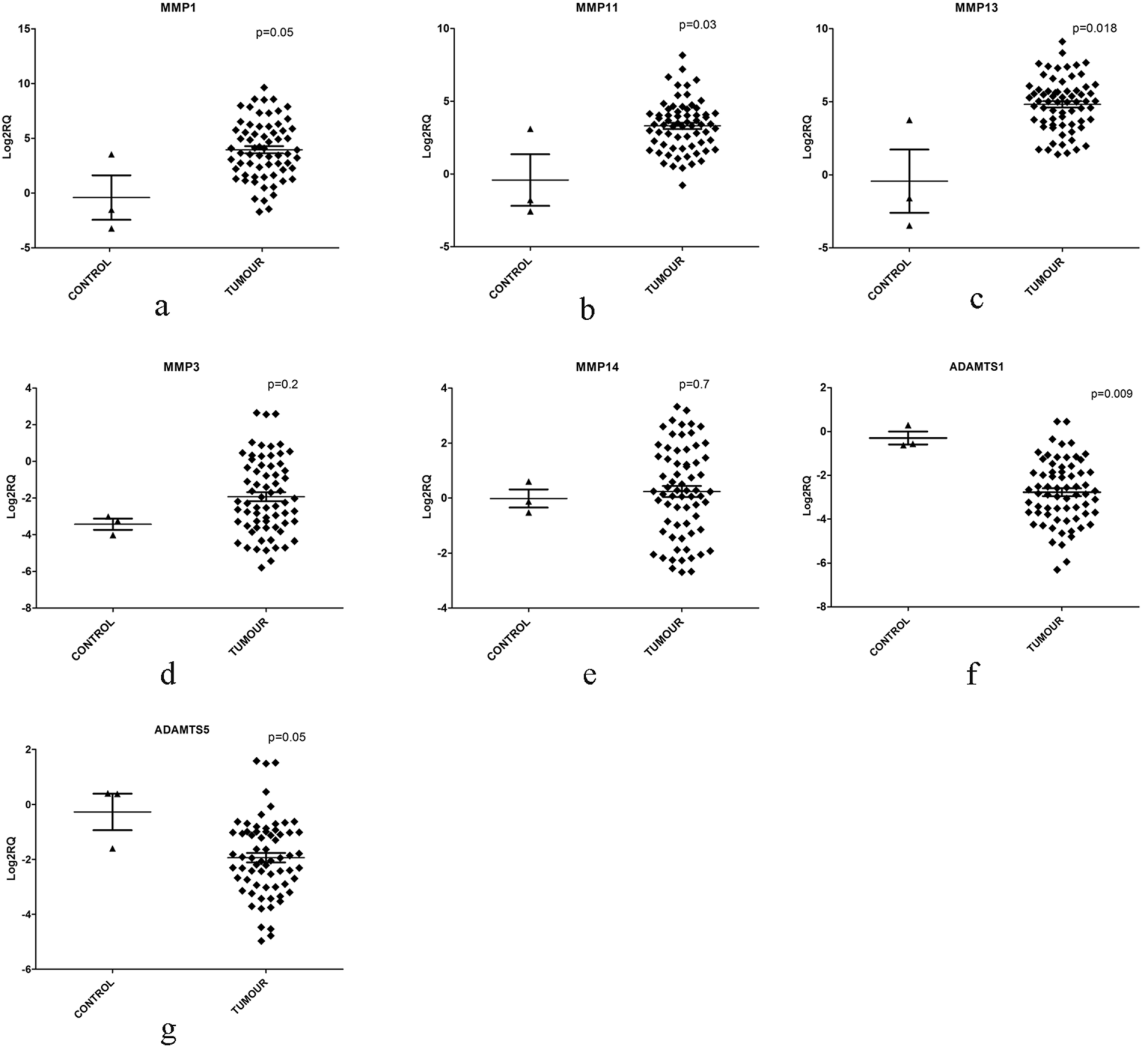
Validation of gene expression of MMPs by quantitative reverse transcription PCR. Scatter plots showing the up-regulation of (**a**) *MMP1* (p = 0.05), (**b**) *MMP11* (p = 0.03), (**c**) *MMP13* (p = 0.018) (**d**) *MMP3* (p = 0.214), (**e**) *MMP14* (p = 0.722) and down-regulation of *ADAMTS1* (p = 0.009), (**g**) *ADAMTS5* (p = 0.05) in breast tumours compared to controls. The values are the mean of log fold change normalized to endogenous controls, along with the standard error (shown by vertical bars) as obtained by Mann-Whitney U test.

Gene expression profiling showed deregulation of several members of metallopeptidase activity/extracellular matrix activity genes in breast tumours and they formed part of top 50 DEGs. Hence we validated gene expression of *MMP1*, *MMP3*, *MMP11*, *MMP13*, *MMP14*, *ADAMTS1* and *ADAMTS5* genes belonging to metallopeptidase activity, using qPCR in 67 tumours. Overexpression of *MMP1*, FC = 15.4 (p = 0.05), *MMP11*, FC = 6.8 (p = 0.03) and *MMP13*, FC = 12.3 (p = 0.018) genes in breast tumours reached statistical significance, compared to their expression in controls (Supplementary Table S16); while, *MMP3* (FC = 6.8, p = 0.214) and *MMP14* (FC = 0.48, p = 0.7) genes did not show specific pattern of expression among tumours. Adamalysins genes/ A distintegrin and metalloproteinase with thrombospondin motifs (ADAMTS) family genes, *ADAMTS1* (FC = −9.4, p = 0.009), and *ADAMTS5* (FC = −5.7, p = 0.05) were significantly down-regulated in tumours, as compared to controls (Supplementary Table S16, Fig. 6). Further, it was found that the down-regulated expression of *ADAMTS5* was significantly associated with LT (FC = −6.5, p = 0.013, Supplementary Table S17). Though expression of various MMP genes was found to be relatively higher in ET compared to LT, it did not reach statistical significance. We further, analysed the differential expression of metallopeptidase genes for their association with various clinicopathological features. Overexpression of *MMP11* gene (p = 0.04) was significantly associated with the metastasis, while overexpression of *MMP1* was associated with loss of ER (p = 0.01), and PR (p = 0.006), on the other hand, overexpression of *MMP13* was found to be associated with overexpression of HER2/neu in patients (p = 0.023). The qPCR data confirmed the overexpression of several MMP genes in breast tumours that was observed using microarray experiments.

## Discussion

Breast cancer incidence is increasing globally (24.2%) as well as in India (15.46%) and has become the most common cancer among Indian women^7,8^. To gain insight into the molecular mechanisms involved in the pathogenesis of breast cancer in Indian women, we have carried out gene expression profiling, wherein we have analysed the gene expression profiles associated with breast tumours and those associated with age and tumour stage. So far, there has been a single report where gene expression profiles of breast tumours were studied in Indian women, however, the authors have analysed gene expression profiles of ER positive and negative tumours, where they have found four hormone-responsive genes as DEGs^11^. To our knowledge, this is the first study describing comprehensive gene expression profiles in Indian women and demonstrating the existence of molecular subtypes using gene expression profiles in breast tumours.

The present study identified 2413 differentially expressed genes comprising of top up-regulated genes such as *COL10A1*, *COL11A1*, *MMP1*, *MMP13*, *MMP11*, *GJB2*, *CST1*, *KIAA1199*, *CEACAM6*, and *BUB1*; top down-regulated genes comprising of *PLIN1*, *FABP4*, *LIPE*, *AQP7*, *LEP*, *ADH1A*, *ADH1B*, *CIDEC*, *THRSP*, *GPD1*, *TIMP4*, and *KIAA1881* (Supplementary Fig. S2, Supplementary Table S2). Among the top DEGs, up-regulated expression of genes such as *COL10A1*, *MMP1*, *MMP11*, and *BUB1*; down-regulated expression of genes such as *ADH1B*, *CIDEC*, *FABP4*, *AQP7*, *RBP4*, *CDO1*, *FIGF*, and *LPL* were reported to be differentially expressed in breast and or other cancers by various authors using microarray profiling in western population^26–38^, showing concordance with the present study. In the present study, we found up-regulation of cell cycle genes such as *BUB1*, *CCNA2*, *CCNB2*, and *CDC2*; up-regulated expression of *BUB1*, *CCNA2*, *CCNB2* and *CDC2* has also been reported in breast^39,40^ and several other cancers^41–46^ using microarray and were found to be associated with poor prognosis of the disease^44,47–49^. Overexpression of the above cell cycle genes may be contributing to the uncontrolled proliferation of the tumour cells and hence may serve as biomarkers and targets for therapy.

Overexpression of genes involved in DNA replication such as *MCM2*, *MCM6*, *MCM10*, and *RAD21* was observed in the present study, increased expression of *MCM*2, *MCM6*, and *MCM10* have been reported in breast and several other epithelial malignancies by transcriptome profiling, and was associated with poor prognosis^50–52^. Furthermore, the focal adhesion genes such as *COL1A1*, *COL10A1*, and *COL11A1* were also found to be up-regulated in the present study and are also reported to be up-regulated in various cancers including breast tumours^37,53–55^. In cancer cells, collagen gene expression is known to increase drug resistance by inhibiting drug penetration as well as cause an increase in apoptosis resistance, thus, in turn, promoting tumour progression^37,56–58^.

In the present study genes such as *PLIN1*, *FABP4*, *LIPE*, *LEP*, *CIDEC*, *THRSP*, *AQP7*, *ADH1A*, *ADH1B*, *GPD1*, and *TIMP4* were found to be down-regulated, involved mainly in lipid metabolism, lipolysis, oxidoreductase activity, and PPAR pathways. In concordance with the above findings, down-regulated expression of lipid metabolism genes such as *LEP*, *CIDEC*, *THRSP*, *PLIN1*, *GPD1*, and *FABP4* genes were also reported at the transcript level by various authors in breast^54,59–64^ and other cancers such as gastric^65^, hepatocellular^66^ and keratoacanthomas^67^. Similar to that observed in the present study, down-regulated expression of aquaporin, *AQP7* gene belonging to water channel family, *TIMP4* belonging to mettalloproteinases inhibitor family member was also reported in breast and hepatocellular carcinoma at transcript level^68–70^. Contrary to the down-regulation observed in the present study, up-regulation of *FABP4*, *LEP*, *CIDEC* genes was reported at transcript and or protein level^35,71–74^ in lung, thyroid, colorectal, and tongue squamous cell carcinoma; these differences may be attributed to tissue-specific differences in gene expression, differences due to the techniques employed in the studies, which need to be established by experimental validation.

Networking analysis was done to identify genes involved in regulation of gene expression in cancer cells, *AURKB*, *CENPA*, *TOP2A*, *BUB1*, *CCNB2*, *MMP1*, and *SPP1* were identified as top hub genes from the up-regulated genes; suggesting these genes might be playing key regulatory role in breast carcinogenesis through deregulation of cell cycle and in invasion/metastasis. Similarly, genes such as *CAV1*, *ACACB*, *NTRK2*, *KLF4*, and *MYH11* were the key down-regulated hub genes suggesting a possible role of their decreased expression in breast carcinogenesis.

Comparison of gene expression profiles of Indian patients with that of western patients led to the identification of 558 genes specifically found to be deregulated in Indian patients, suggesting some differences in the gene sets between these populations. The differences in DEGs among the two populations may be partly due to differences in platforms, experimental procedures or genetic makeup of the populations. Up-regulated expression of *COL10A1*, *MMP11*, *CST1*, *GJB2*, *MMP1*, *MMP13*, and *CEACAM6*; down-regulated expression of *ADH1B*, *CIDEC*, *THRSP*, *GPD1*, *TIMP4*, *FABP4*, and *SCARA5* genes was common in breast cancers of the two populations. The similarity in the DEGs between the Indian and western patients suggests a similarity in the molecular events associated with breast carcinogenesis. Further, we compared DEGs obtained in the present study with that of Lebanese population^54^, where several genes were found to be common between the two populations. Up-regulated expression of *COL11A1*, *GJB2*, *MMP13*, *EPYC*, *CEP55*, and *MELK* and down-regulated expression of *PLIN*, *TIMP4*, *LEP*, *LYVE1*, *SDPR*, *FIGF*, and *LPL* was observed in common with the Lebanese population from the top 50 genes found in the present study. This is pointing towards a possible existence of greater similarity in the molecular pathogenesis of breast cancers amongst the Asian population compared to the western population.

Comparison of expression profiles of ET (≤40 years) and LT (≥55 years) yielded few genes that are unique between the two groups, 7 genes *B4GALNT1*, *S100P*, *KLK4*, *HIST3H2A*, *DRD4*, *PCSK1N*, and *BAPX1* were significantly overexpressed in early-onset tumours compared to late-onset tumours. Overexpression *B4GALNT1* causes deregulation of glycosphingolipid biosynthesis and is reported to be up-regulated in breast cancer stem cells^75^ at the transcript level, similarly, *S100P*^76^, *KLK4*^77,78^, *DRD4*^79^, and *BAPX1*^80^ genes were also reported to be up-regulated in breast and other cancers at mRNA level. Several of these genes are known to induce invasion and metastasis^81–84^, breast cancer in young patients is known to be aggressive^85–88^, the overexpression of these genes may be thus contributing to the aggressive behavior of the early-onset cancers. Anders *et al*.^89–91^ have analysed gene expression profiles between early-onset patients and late-onset patients, where 693 DEGs were found initially, later the significance was lost when they have corrected the gene differences for subtypes and for ER and histological grades. However, such segregated analysis could not be carried out in the present study due to the small sample size. Further, we compared gene expression profiles between lower and advanced stages of tumours; we identified 200 genes uniquely deregulated in advanced stage cancers, involving pathways such as cell adhesion (*VCAN*), ECM receptor interaction (*COL1A2*, *COL3A1*, *ITGA11*, and *TNN*) and pathways in cancer (*JUN*, and *MYC*) which may be contributing to increased proliferation, migration and increased angiogenesis in advanced stage of tumours.

Molecular subtyping and hierarchical clustering of gene expression profiles of breast tumours using PAM50 molecular signatures, yielded distinct clusters corresponding to each molecular subtype, showing the existence of molecular subtypes in these patients. Among these patients, 44% tumours falling into luminal subtype, 31% into basal subtype and 20% into HER2/neu overexpressing subtype, and 3% into normal-like subtype, which is more or less similar to that reported in the western population^28,29^. To our knowledge this is the first study acknowledging the existence of molecular subtypes from the Indian subcontinent based on gene expression profiles; earlier, Kumar *et al*.^92^ reported the existence of molecular subtypes based on the expression of ER, PR, HER2/neu and cytokeratins at protien level, however, transcriptome-based subtyping has not been demonstrated so far.

Matrix metalloproteinases (MMPs) belong to Zn2+-dependent endopeptidases family, capable of catalyzing the hydrolysis of collagen, forming a major part of extracellular matrix (ECM) remodelling^93,94^. Metallopeptidases genes were one of the functional class of genes found to have deregulated expression in breast cancers in the present study and hence we validated some of the genes by qPCR. QPCR analysis confirmed the up-regulated expression of *MMP1*, *MMP13*, and *MMP11* genes and down-regulated expression of *ADAMTS1* and *ADAMTS5* genes in breast cancers observed in the microarray experiments. Overexpression of *MMP1*, *MMP11*, and *MMP13* was also reported in breast^95^ and several other cancers such as gastric, oral, colorectal, oesophageal and nasopharyngeal at the transcript and or protein level^30,54,96–103^. Upregulated expression of MMPs has been reported in cancer, vascular diseases and many different types of inflammatory diseases^104^, their overexpression results in increased invasion and metastasis in cancer cells^105^. *A disintegrin and metalloproteinase with thrombospondin motif* (*ADAMTS*), superfamily genes play, an important role in ECM assembly and degradation, several of them act as tumour or metastasis suppressors by influencing cell proliferation, migration, apoptosis, and angiogenesis^106^. In the present study, *ADAMTS1* and *ADAMTS5* genes were observed to be underexpressed, down-regulated expression and antitumour acitivity of these genes were reported in breast cancers^107^ and gastric carcinoma carcinoma^108^ respectively at both transcript and protein. An association between overexpression of *MMP1* transcripts, with loss of ER (p = 0.01), and PR (p = 0.006) was found in the present study, Nakopoulou *et al*.^109^ has also found a similar inverse association with PR expression at the protein level, supporting findings of the present study. Further, overexpression of *MMP13* was found to be associated with overexpression of HER2/neu in patients (p = 0.023), Zhang *et al*.^110^ have also reported similar association in breast cancer with protein level expression. Interestingly, down-regulation of *ADAMTS5* was found to be associated with late-onset tumours (≥55 years) compared to ET (≤40 years), (FC = −6.5 in LT and FC = −4.5 in ET, p = 0.013), suggesting the involvement of loss of this gene in the molecular pathogenesis with late-onset breast cancer. To our knowledge, the down-regulated expression of *ADAMTS5* in breast tumours and its association with late-onset breast cancer (old age of patient) is reported for the first time in the present study. Together, deregulated expression of these matrix remodeling factors in breast tumours may be contributing to the degradation of ECM and invasion and metastasis in breast tumours, suggesting a pivotal role played by these genes in breast tumorigenesis. However, up-regulation of *MMP3* and *MMP14* genes didn’t reach statistical significance, unlike found in microarray data. The discrepancy between microarray and qPCR data could be due to a different number of samples analysed by each method, to some extent, tumour heterogeneity might have also contributed to such differences.

The present study describes comprehensive gene expression profiles of breast tumours from Indian women and the presence of molecular subtypes in this population. Genes involved in cell cycle, ECM, metastasis were some of the essential pathways found to be up-regulated, on the other hand genes involved in lipid metabolism, PPAR were some of the pathways that were found down-regulated. Genes belonging to cell adhesion, cell cycle, ECM receptor interaction pathways were deregulated in early-onset breast cancers. This study confirmed the presence of molecular subtypes in breast tumours based on gene expression profiles, for the first time from Indian patients. Comparison with western data has revealed the presence of several deregulated genes that are common between Indian and western patients suggesting a similarity in the molecular mechanisms; however, a higher similarity was with that of the Asian population. Comparison of gene expression profiles in early- and late-onset tumours showed several common DEGs between the groups, but with differences in fold change of their gene expression. Further, significant down-regulation of *ADAMTS5* in old age patients had been reported for the first time in breast cancer patients.

### Limitations

The current study describes gene expression profiles from Indian breast cancer patients, yet it has some limitations. In the present study, we have analysed gene expression profiles of 29 tumours and 9 controls, however, to extrapolate this outcome to the breast cancer patients in the Indian subcontinent, gene expression profiles in larger patients set covering various geographical regions in India is warranted. Another limitation of the study is that genes which were found to be differentially expressed in microarray and qPCR, could not be validated at the protein level due to limited resources.

## Supplementary information

Final revised supplementary tables document

Final revised supplementary tables sheet

Supplementary figure
