# Supplementary material for: Study of Gene Expression Profiles of Breast Cancers in Indian Women
Source: Sci Rep. 2019 Jul 10;9:10018. doi: 10.1038/s41598-019-46261-1 (PMC6620270; doi:10.1038/s41598-019-46261-1)
Supplement: Supplementary file 1 — Final revised supplementary tables document [file 41598_2019_46261_MOESM1_ESM.docx]

**Study of Gene Expression Profiles of Breast cancers in Indian Women**

**Shreshtha Malvia**^†^**^1^, Sarangadhara Appala Raju Bagadi^†^*^1^, Dibyabhaba Pradhan^2^, Chintamani Chintamani^3^, Amar Bhatnagar^4^, Deepshikha Arora^5^, Ramesh Sarin^6^, Sunita Saxena*****^1^**

| **AGE** | **Number of cases (%)** |
| --- | --- |
| ≤40 | 35 (45.45%) |
| ≥55 | 42 (54.54%) |
|  |  |
| **Menopausal Status** | |
| Premenopausal | 35 (45.45%) |
| Postmenopausal | 42 (54.54%) |
|  |  |
| **Tumour Stage** | |
| Stage I | 2 (2.59%) |
| Stage II | 32 (41.5%) |
| Stage III | 37 (48.05%) |
| Stage IV | 3 (3.89%) |
| Undetermined | 3 (3.89%) |
|  |  |
| **IHC Subtypes** | |
| Luminal | 33 (42.85%) |
| Basal | 17 (22.07%) |
| HER2/neu overexpressing | 23 (29.87%) |
| Undetermined | 4 (5.19%) |
|  |  |
| **Estrogen Receptor** | |
| Positive | 33 (42.85%) |
| Negative | 40 (51.94%) |
| Undetermined | 4 (5.19%) |
|  |  |
| **Progesterone Receptor** | |
| Positive | 29 (37.66%) |
| Negative | 44 (57.14%) |
| Undetermined | 4 (5.19%) |
|  |  |
| **HER2/neu overexpressing** | |
| Positive | 39 (42.85%) |
| Negative | 34 (44.15%) |
| Undetermined | 4 (5.19%) |
|  |  |
| **Chemotherapy/ Radiotherapy received** | |
| Yes | 0 |
| No | 77 (100%) |

**Supplementary Table S2:** Number of patients belonging to different breast cancer groups

| **Genes** | **Primer sequence** |
| --- | --- |
|  |  |
| MMP1_F | CGAATTTGCCGACAGAGATG |
| MMP1_R | AAGCCAAAGGAGCTGTAGAT |
|  |  |
| MMP3_F | CAGGCTTTCCCAAGCAAATAG |
| MMP3_R | CCAACTGTGAAGATCCAGTAAAG |
|  |  |
| MMP11_F | TGGGTCCTGACTTCTTTGG |
| MMP11_R | CATGGGTCTCTAGCCTGATATT |
|  |  |
| MMP13_F | TTGTTGCTGCGCATGAGTT |
| MMP13_R | TTGCCGGTGTAGGTGTAGATAG |
|  |  |
| MMP14_F | CGGCCTTCTGTTCCTGATAA |
| MMP14_R | GCTCCTTGAAGACAAACATCTC |
|  |  |
| ADAMTS1_F | CTTCACCACAGCCCATGAATTAG |
| ADAMTS1_R | GGAATCCTGGTTCACACCATTAAG |
|  |  |
| ADAMTS5_F | CGCTAAGACACAGCCAATAATC |
| ADAMTS5_R | TCCCTCAGAAGTTGTGTGTAG |
|  |  |
| 18S_F | GGATCCATTGGAGGGCAAGT |
| 18S_R | CCCAAGATCCAACTACGAGCTT |
|  |  |
| ACTINB_F | ATGCTTCTAGGCGGACTATG |
| ACTINB_R | AGCCATGCCAATCTCATC |
|  |  |
| PSMC4_F | ATCTGTCAGGAGAGTGGAATG |
| PSMC4_R | CCTTCTTGATGACAGTCTTGTATG |

**Supplementary Table S3:** List of primers and their corresponding sequences used for qPCR

| S.No. | Pathway Name | Impact Factor | Input Genes in Pathway | List of Genes | Adjusted p-value |
| --- | --- | --- | --- | --- | --- |
| 1 | Cell cycle | 26.56 | 29 | *E2F2, E2F3, CDC2, E2F5, PKMYT1, TTK, CDCA5, CHEK1, PTTG1, CCNE2, CCNE1, RAD21, MCM7, BUB1, CCNA2, CDC20, ESPL1, MCM2, CDC25C, MCM4, CDC25A, MCM6, CCNB1, MAD2L1, CCNB2, PLK1, GSK3B, BUB1B,*  and *CCNB1* | 1.38E-11 |
| 2 | Systemic lupus erythematosus | 23.226 | 24 | *HIST2H2AA3, HIST1H2BC, HIST1H2BD, HIST1H4K, HIST1H2BE, HIST1H2BF, HIST1H2BG, HIST1H2BH, HIST2H4A, HIST2H2AB, HIST1H2BK, CD80, FCGR1A, HIST2H2AC, HIST1H2BJ, HIST1H4E, SNRPB, H2AFZ, HIST1H3D, H2AFX, HIST1H3F, HIST1H2AM, HIST1H3H,* and *HIST1H4H* | 3.09E-10 |
| 3 | DNA replication | 20.508 | 12 | *DNA2, RFC4, MCM7, POLE2, RFC2, PRIM2, MCM2, POLA2, RNASEH2A, MCM4, FEN1,* and *MCM6* | 3.23E-09 |
| 4 | ECM-receptor interaction | 10.292 | 11 | *COL3A1, COL1A2, ITGA11, COL1A1, COL24A1, COL11A1, COL5A2, COL5A1, SPP1, FN1,* and *HMMR* | 3.65E-04 |
| 5 | p53 signaling pathway | 8.15 | 9 | *CCNB1, CCNE2, CCNE1, CCNB2, RRM2, BAX, CHEK1, GTSE1,* and *CDC2* | 0.001501 |

**Supplementary Table S5:** Gene ontology analysis of up-regulated DEGs in breast tumours

| S.No. | Pathway Name | Impact Factor | Input Genes in Pathway | List of Genes | Adjusted p-value |
| --- | --- | --- | --- | --- | --- |
| 1 | Leukocyte trans endothelial migration | 398.428 | 15 | *MYL7, CLDN19, GNAI1, CLDN5, CLDN11, CXCL12, PXN, CDH5, MYL9, CLDN15, ARHGAP5, PECAM1, ESAM, RAPGEF3,* and *JAM2* | 0.00209 |
| 2 | Cell adhesion molecules (CAMs) | 243.355 | 18 | *SELP, CADM3, ITGA7, PTPRM, C10ORF54, ICAM2, CLDN5, NLGN1, CLDN11, CDH5, CLDN15, ITGA9, CD34, PECAM1, ESAM, JAM2, NEGR1,* and *PVRL3* | 3.49E-04 |
| 3 | Adherens junction | 30.176 | 11 | *PTPRB, PTPRM, WASF2, SORBS1,* PVRL3*, CDH5, PRR4, CADM3, ANG, TCF7L2,* and *CDH23* | 0.003167 |
| 4 | Complement and coagulation cascades | 19.592 | 17 | *C7, A2M, F10, F13A1, C6, SERPING1, C4BPA, VWF, THBD, SERPINF2, F3, CD59, CFH, TFPI, CFI, CFD,* and *PROS1* | 1.61E-07 |
| 5 | PPAR signaling pathway | 18.494 | 17 | *LPL, EHHADH, RXRA, PLIN PPARG, AQP7, ACADL, ADIPOQ, PCK1, ACSL1, CD36, SORBS1, FABP4, PLTP, ANGPTL4, NR1H3, and PPARGC1A* | 1.61E-07 |
| 6 | Circadian rhythm | 15.269 | 3 | *PER1, CRY2,* and *RXRA* | 0.033661 |
| 7 | Focal adhesion | 13.052 | 26 | *CAV2, MYL7, CAV1, TLN2, FIGF, IGF1, FLNC, PXN, MYL9, LAMA2, VWF, VEGFC, ITGA9, LAMA4, LAMA3, LAMB2, ARHGAP5, LOXL4, CCND2, JUN, ITGA7, RELN, TNN, PDGFD, MYLK,* and *THBS4* | 6.02E-05 |
| 8 | Adipocytokine signaling pathway | 11.879 | 12 | *LEP, IRS2, ACSL1, CD36, SOCS3, LEPR, RXRA, ACACB, POMC, ADIPOQ, PPARGC1A, and PCK1* | 3.37E-04 |
| 9 | Tight junction | 10.153 | 15 | *JAM2, MPDZ, JUN, CLDN11, CLDN15, CLDN19, CLDN5, ITGB1BP1, ACTG2, RAPGEF3, MYL9, MYLK7, MYLK, SLC9A3,* and *RAPGEF3* | 0.007214 |

**Supplementary Table S6:** Gene ontology analysis of down-regulated DEGs in breast tumours

| **Gene Name** | **(FC)** | **Adjusted p-value** | **Accession** |
| --- | --- | --- | --- |
| *COL10A1* | 23.2815 | 5.96E-06 | NM_000493.2 |
| *MMP11* | 15.45492 | 6.55E-06 | NM_005940.3 |
| *GJB2* | 13.68954 | 8.27E-06 | NM_004004.3 |
| *CST1* | 11.94889 | 0.004912 | NM_001898.2 |
| *MMP1* | 9.096327 | 0.008953 | NM_002421.2 |
| *MMP13* | 7.297567 | 0.002203 | NM_002427.2 |
| *CEACAM6* | 7.014203 | 0.042617 | NM_002483.3 |
| *BUB1* | 6.795974 | 9.11E-05 | NM_004336.2 |
| *ASPM* | 6.500159 | 2.39E-05 | NM_018136.2 |
| *FAM83D* | 6.454691 | 0.000158 | NM_030919.1 |
| *C2ORF40* | -21.1401 | 2.51E-06 | NM_032411.1 |
| *SCARA5* | -21.6551 | 2.80E-06 | NM_173833.3 |
| *G0S2* | -22.0594 | 3.68E-07 | NM_015714.2 |
| *C7* | -22.871 | 1.39E-05 | NM_000587.2 |
| *FABP4* | -23.8769 | 0.000104 | NM_001442.1 |
| *TIMP4* | -24.1717 | 1.03E-07 | NM_003256.2 |
| *GPD1* | -24.9122 | 3.83E-10 | NM_005276.2 |
| *THRSP* | -26.7033 | 3.01E-05 | NM_003251.2 |
| *CIDEC* | -27.4842 | 3.18E-08 | NM_022094.2 |
| *ADH1B* | -33.9347 | 3.60E-08 | NM_000668.3 |

**Supplementary Table S7:** List of top DEGs commonly deregulated in Indian and western datasets (FC ≥2, and adjusted p-value ≤0.05)

| **Gene Name** | **(FC)** | **Adjusted p- value** | **Accession** |
| --- | --- | --- | --- |
| *KIAA1199* | 9.509539 | 0.000351 | NM_018689.1 |
| *CKAP2L* | 6.264448 | 9.48E-05 | NM_152515.2 |
| *TUBB3* | 6.044887 | 0.00046 | NM_006086.2 |
| *CENPA* | 5.311936 | 5.86E-05 | NM_001809.2 |
| *CDC45L* | 4.832954 | 0.001179 | NM_003504.3 |
| *C6ORF126* | 4.722761 | 0.028214 | NM_207409.1 |
| *PPAPDC1A* | 4.614044 | 0.000137 | NM_001030059.1 |
| *KLK4* | 4.404234 | 0.020902 | NM_004917.2 |
| *CDC2* | 4.340372 | 0.000164 | NM_033379.2 |
| *PTTG3P* | 4.210745 | 0.000407 | NR_002734.1 |
| *FIGF* | -10.6485 | 1.73E-07 | NM_004469.2 |
| *SAA2* | -11.9734 | 0.000866 | NM_030754.2 |
| *XLKD1* | -13.1265 | 2.60E-06 | NM_006691.2 |
| *DARC* | -15.1034 | 0.000104 | NM_002036.2 |
| *AQP7* | -16.3904 | 3.36E-10 | NM_001170.1 |
| *AQP7P2* | -18.3621 | 3.36E-10 | XR_001073.1 |
| *SAA1* | -18.744 | 3.74E-05 | NM_000331.2 |
| *ADH1A* | -46.9269 | 1.12E-05 | NM_000667.2 |
| *KIAA1881* | -48.6453 | 2.71E-07 | Hs.567652 |
| *PLIN* | -49.7345 | 3.12E-08 | NM_002666.3 |

**Supplementary Table S8:** List of top DEGs in Indian dataset which were not in common with the western dataset (FC ≥2, and adjusted p-value ≤0.05)

| S.No. | Pathway Name | Impact Factor | Input Genes in Pathway | List of Genes | Adjusted p-value |
| --- | --- | --- | --- | --- | --- |
| 1 | Leukocyte trans endothelial migration | 337.652 | 15 | *MYL7, GNAI1, CLDN5, CLDN11, PXN, CDH5, CLDN15, ARHGAP5, PECAM1, ESAM, RAPGEF3, JAM2, F11R, MAPK13,* and *CLDN19* | 0.009856 |
| 2 | Cell adhesion molecules (CAMs) | 197.462 | 19 | *SELP, CADM3, CLDN19, CDH15, PVRL2, ICAM2, CLDN5, NLGN1, CLDN11, CDH5, CLDN15, ITGA9, CD34, F11R, PECAM1, ESAM, JAM2, VCAN,* and *PVRL3* | 9.69E-04 |
| 3 | PPAR signaling pathway | 16.06 | 17 | L*PL, ACADM, OLR1, PLIN, EHHADH, PPARG, AQP7, ACADL, ADIPOQ, MMP1, PCK1, ACSL1, CD36, SORBS1, FABP4, ANGPTL4,*  and *NR1H3* | 1.72E-06 |
| 4 | Cell cycle | 15.976 | 24 | *E2F2, CDC14B, PKMYT1, SMAD3, TTK, CHEK1, CDC20, ESPL1, PTTG1, CDC25C, MCM4, CCNB1, CDKN1C, CCNE2, CCNE1, CDKN2A, CCNB2, PLK1, CDKN2C, GSK3B, BUB1, CCNA2, CDC2,* and *CDC45L* | 3.72E-07 |
| 5 | ECM-receptor interaction | 15.108 | 19 | *IBSP, COL3A1, ITGA11, COL5A2, COL5A1, HMMR, LAMA2, VWF, CD36, LAMA3, ITGA7, COL1A2, RELN, SV2B, COL1A1, COL24A1, COL11A1, SPP1,* and *FN1* | 1.50E-06 |
| 6 | Pathways in cancer | 12.634 | 43 | *E2F2, ADCY4, HRAS, FGF7, GNAI1, STAT5A, STAT5B, PPARG, GNG11, ZBTB16, KIT, TCF7L2, MMP1, CCNE2, WNT2, CCNE1, FOS, CDKN2A, ACVR1C, FGF2, EVI1, FN1, PIK3R2, CEBPA, IL6, BMP2, PTGER4, BRAF, FIGF, TGFBR2, LEF1, IGF1, SMAD3, BIRC5, FZD2, DAPK2, FZD4,LAMA2, LAMA3, JUN, GSK3B, CKS2,* and *WNT11* | 1.94E-05 |

**Supplementary Table S11:** Gene ontology analysis of DEGs in early-onset tumours

| S.No. | Pathway Name | Impact Factor | Input Genes in Pathway | List of Genes | Adjusted p-value |
| --- | --- | --- | --- | --- | --- |
| 1 | Leukocyte trans endothelial migration | 398.836 | 19 | *ARHGAP5*, *CDH5*, *CLDN11*, *CLDN5*, *CXCL12*, *ESAM*, *GNAI1*, *JAM2*, *JAM3,* *MAPK13*, *MYL7*,*MYL9*, *PECAM1*,*PXN*, *RAPGEF3*, *CLDN19*, *ICAM2,* *GNAI1*, and *CLDN15* | 0.011403 |
| 2 | Cell adhesion molecules (CAMs) | 273.856 | 20 | *CADM3*, *LAMB2, CD80, CD34, CDH5*, *CLDN11*, *CLDN5*, *ESAM*, *ICAM2*, *JAM2*, *LAMA4,* *ITGA7,* *ITGA9, NLGN1*, *CMA1*, *PCOLCE2,* *PECAM1*, *PTPRM*, *PVRL3*, and *SELP* | 0.018652 |
| 3 | PPAR signalling pathway | 16.049 | 20 | *SLC27A1, LPL, OLR1, EHHADH, RXRA, PPARG, AQP7, ACADL, ADIPOQ, MMP1, PCK1, ACSL1, CD36, SORBS1, FABP4, GK, PLTP, ANGPTL4, NR1H3,*  and *PLIN* | 2.85E-06 |
| 4 | Cell cycle | 27.716 | 38 | *E2F2, E2F3, CDC2, E2F5, CDC14B, PKMYT1, TTK, PRKDC, CHEK1, PTTG1, CCNE2, CCNE1, RAD21, MCM7, CDKN2C, BUB1, CCNA2, RBL1, ESPL1, CDC20, MCM2, CDC25C, MCM4, CDK2, CDC25A, MCM6, CDKN1C, CCNB1, CCNB2, MAD2L1, CCND2, PLK1, GSK3B, PCNA, BUB1B, GADD45B, ORC1L,* and *CDC45L* | 2.79E-12 |
| 5 | ECM-receptor interaction | 12.463 | 21 | *COL3A1, COL5A2, COL5A1, HMMR, LAMA2, VWF, LAMA4, LAMB2, CD36, LAMA3, ITGA7, COL1A2, RELN, TNN, SV2B, COL1A1, COL24A1, COL11A1, SPP1, FN1,* and *THBS4* | 1.77E-05 |
| 6 | Adherens junction | 26.362 | 16 | *PTPRB, PTPRJ, PTPRM, WASF3, NLK, WASF2, TGFBR2, LEF1, TCF7L2, TCF7L1, CSNK2A1, SORBS1, MAPK3, SSX2IP, ACVR1C,* and *PVRL3* | 0.001502 |
| 7 | Pathways in cancer | 11.622 | 54 | *ADCY4, E2F2, E2F3, FGF7, STAT5A, ADCY6, STAT5B, PPARG, FOXO1, CXCL12, MMP1, CCNE2, WNT2, FOS, EDNRB, CCNE1, WNT3, GNG2, FGF2, PTGER2, PTGER4, BRAF, RXRA, CYCS, LEF1, DAPK2, CDK2, VEGFC, LPAR5, JUN, MAPK3, WNT11, GNAI1, GRB2, GNG11, KIT, ZBTB16, TCF7L2, TCF7L1, LAMB2, PIK3R2, MSH6, BMP2, IL6, EPAS1, TGFBR2, IGF1, FZD5, FZD4, LAMA2, WNT7B, LAMA4, LAMA3,* and *GSK3B* | 4.05E-05 |

**Supplementary Table S12:** Gene ontology analysis of DEGs in late-onset tumours

| **GENE** | **(FC)-Microarray** | **Adjusted p-value** | **(FC)-qPCR** | **Adjusted p-value** |
| --- | --- | --- | --- | --- |
| ADAMTS1 | -5.5308 | 4.48E-06 | -9.4051 | 0.009 |
| ADAMTS5 | -3.4743 | 0.00089 | -5.6627 | 0.05 |
| MMP1 | 9.09633 | 0.00895 | 15.4261 | 0.05 |
| MMP11 | 15.4549 | 6.55E-06 | 6.82081 | 0.03 |
| MMP13 | 7.29757 | 0.0022 | 12.3449 | 0.018 |
| MMP14 | 2.51886 | 0.00616 | 0.48461 | 0.722 |
| MMP3 | 4.82262 | 0.01643 | 6.68377 | 0.214 |

**Supplementary Table S16:** Fold expression of MMP genes obtained by microarray and qPCR in breast tumours

| **GENE** | **(FC)-ET** | **(FC)-LT** | **p-value** |
| --- | --- | --- | --- |
| ADAMST1 | -7.76766 | -10.9943 | 0.25 |
| ADAMST5 | -4.54458 | -6.74784 | 0.013 |
| MMP1 | 17.52682 | 13.44894 | 0.603 |
| MMP11 | 7.807872 | 5.862773 | 0.554 |
| MMP13 | 14.96795 | 9.799033 | 0.985 |
| MMP14 | 0.542113 | 0.428789 | 0.985 |
| MMP3 | 7.284474 | 6.10074 | 0.669 |

**Supplementary Table S17:** Fold expression of MMP genes obtained by qPCR in early- and late-onset breast tumours

**Legends for Supplementary Tables (in excel)**

**Supplementary Table S1:** Supplementary Table S1: Clinicopathological details of patient samples used in microarray and or quantitative PCR, [microarray (M), quantitative PCR (qPCR), invasive ductal carcinoma (IDC), negative (N), positive (P), details not available (NA)]

**Supplementary Table S4:** List of Differentially expressed genes identified by microarray (FC ≥±1.5, and adjusted p-value ≤0.05) in breast tumours

See Supplementary Table S4

**Supplementary Table S9**: List of DEGs found in early-onset tumours (FC ≥±1.5, and adjusted p-value ≤0.05)

See Supplementary Table S9

**Supplementary Table S10:** List of DEGs found in late-onset tumours (FC ≥±1.5, and adjusted p-value ≤0.05)

See Supplementary Table S10

**Supplementary Table S13:** List of DEGs found in lower stage tumours (FC ≥±1.5, and adjusted p≤0.05)

See Supplementary Table S13

**Supplementary Table S14:** List of DEGs found in advanced stage tumours (FC ≥±1.5, and adjusted p≤0.05)

See Supplementary Table S14

**Supplementary Table S15:** List of genes associated with different molecular subtypes, which were identified in the Indian population using 'Genefu' package

See Supplementary Table S15
