# Supplementary material for: Study of Gene Expression Profiles of Breast Cancers in Indian Women
Source: Sci Rep. 2019 Jul 10;9:10018. doi: 10.1038/s41598-019-46261-1 (PMC6620270; doi:10.1038/s41598-019-46261-1)
Supplement: Supplementary file 3 — Supplementary figure [file 41598_2019_46261_MOESM3_ESM.pdf]

Study of Gene Expression Profiles of Breast cancers in Indian Women

Shreshtha Malvia[†1], Sarangadhara Appala Raju Bagadi[†*1], Dibyabhaba Pradhan[2], Chintamani Chintamani[3], Amar Bhatnagar[4], Deepshikha Arora[5], Ramesh Sarin[6], Sunita Saxena[*1]

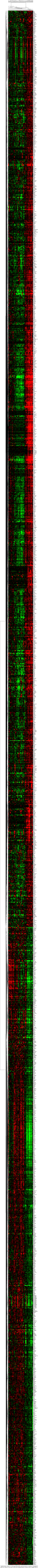

Supplementary Figure S1: Showing differentially expressed genes in tumours. Up-regulated genes are shown in red colour while down-regulated genes in green colour. Sample IDs are shown on x-axis; -ET-early-onset tumour, LT-late-onset tumour, EN-early normal, LN-late normal, y-axis- differentially expressed genes.

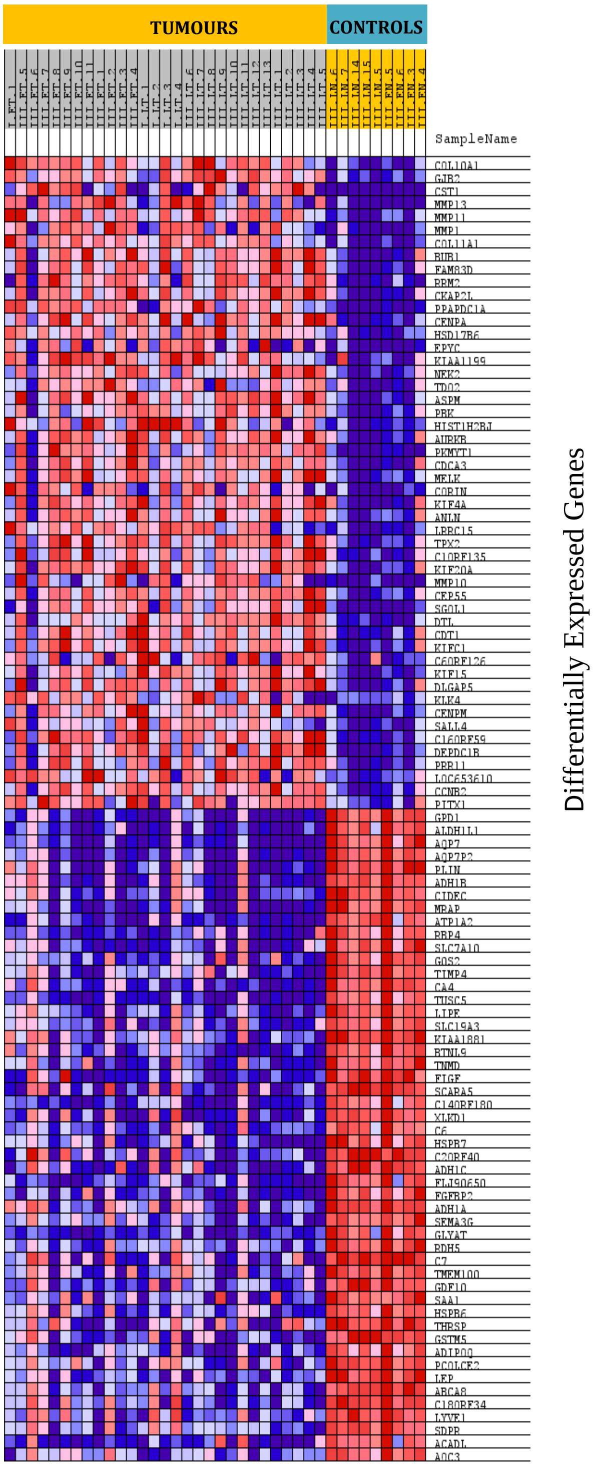

Supplementary Figure S2: Heatmap of the top 50 genes that are differentially expressed in breast tumors obtained after enrichment analysis using GSEA. The up-regulated genes are shown in red colour while down-regulated genes in blue colour. Sample IDs are shown on x-axis; -ET-early-onset tumour, LT-late-onset tumour, EN-early normal, LN-late normal, y-axis- differentially expressed genes.

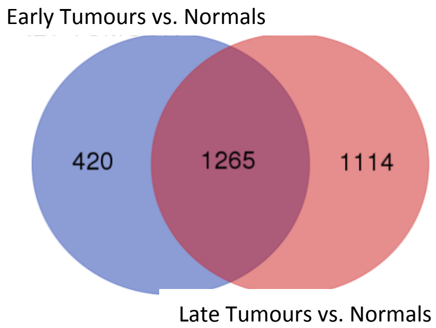

Supplementary Figure S3: Venn diagram showing genes that are common and unique between early- and late-onset tumour groups.

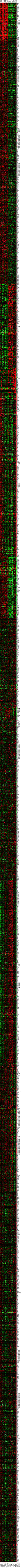

Supplementary Figure S4: Showing clusters of different molecular subtypes in Indian breast cancer patients, obtained using PAM50 classifier. Subtypes are mentioned on x-axis, differentially expressed genes are on y-axis.
